# AFP and eGFR are related to early and late recurrence of HCC following antiviral therapy

**DOI:** 10.1186/s12885-021-08401-7

**Published:** 2021-06-14

**Authors:** Takao Watanabe, Yoshio Tokumoto, Kouji Joko, Kojiro Michitaka, Norio Horiike, Yoshinori Tanaka, Fujimasa Tada, Yoshiyasu Kisaka, Seiji Nakanishi, Kazuhiko Yamauchi, Hironori Ochi, Atsushi Hiraoka, Sen Yagi, Atsushi Yukimoto, Masashi Hirooka, Masanori Abe, Yoichi Hiasa

**Affiliations:** 1 Department of Gastroenterology and Metabology, Ehime University Graduate School of Medicine, Shitsukawa, Toon, Ehime 791-0295 Japan; 2 Center for Liver-Biliary-Pancreatic Diseases, Matsuyama Red Cross Hospital, 1 Bunkyocho, Matsuyama, Ehime 790-8524 Japan; 3 Department of Gastroenterology, Ehime Prefectural Central Hospital, 83 Kasugamachi, Matsuyama, Ehime 790-0024 Japan; 4 Department of Gastroenterology, Saiseikai Imabari Hospital, 7-1-6 Kitamura, Imabari, Ehime 799-1502 Japan; 5 Department of Gastroenterology, Matsuyama Shimin Hospital, 2-6-5 Ootemachi, Matsuyama, Ehime 790-0067 Japan; 6 Department of Internal Medicine, Saiseikai Matsuyama Hospital, 880-2 Yamanishicho, Matsuyama, Ehime 791-8026 Japan; 7 Department of Gastroenterology, Uwajima City Hospital, 1-1 Gotenmachi, Uwajima, Ehime 798-8510 Japan; 8 Department of Gastroenterology, Ehime Prefectural Imabari Hospital, 4-5-5 Ishiicho, Imabari, Ehime 794-0006 Japan; 9 Department of Gastroenterology, National Hospital Organization Ehime Medical Center, 366 Yokogawara, Toon, Ehime 791-0203 Japan

**Keywords:** DAA, Time of recurrence, Renal function, Treatment history, Risk factor, Prediction, Follow-up, HCV, Sex, Male, SVR, Number of treatments, Diabetes mellitus

## Abstract

**Background:**

An unexpected recurrence of hepatocellular carcinoma (HCC) sometimes occurs in patients with hepatitis C virus (HCV) after treatment with direct-acting antivirals (DAAs). However, the characteristics of patients with HCC recurrence may differ depending on time after DAA treatment. We aimed to identify risk factors related to HCC recurrence according to time after DAA treatment.

**Methods:**

Of 1663 patients with HCV treated with a DAA, 199 patients had a previous history of HCC. We defined HCC recurrence within 1 year after DAA treatment as ‘early recurrence’, and recurrence more than 1 year after as ‘late recurrence’. The different risk factors between the early and late phases of HCC recurrence after the end of DAA therapy were investigated.

**Results:**

Ninety-seven patients experienced HCC recurrence during the study period. Incidences of recurrence were 29.8, 41.0, and 53.4% at 1, 2, and 3 years, respectively, after the end of DAA therapy. Multivariate analysis identified post-treatment α-fetoprotein (AFP) as an independent factor contributing to HCC recurrence in the early phase (hazard ratio, 1.056; 95% confidence interval, 1.026–1.087, *p* < 0.001) and post-treatment estimated glomerular filtration rate (eGFR) (hazard ratio, 0.98; 95% confidence interval, 0.96–0.99, *p* = 0.032) as a predictor of HCC recurrence in the late phase.

**Conclusion:**

Patients with higher post-treatment AFP in the early phase and those with lower post-treatment eGFR in the late phase had a high risk of HCC recurrence. The risk factors associated with HCC recurrence after DAA treatment were different between the early and late phases.

**Supplementary Information:**

The online version contains supplementary material available at 10.1186/s12885-021-08401-7.

## Introduction

Chronic hepatitis C virus (HCV) infection is a leading cause of cirrhosis, liver failure, and hepatocellular carcinoma (HCC) [1–3]. In addition, HCV infection may promote carcinogenesis. Therefore, the final goal of HCV treatment is eradication of the virus to prevent HCC and/or liver-related death. For decades, interferon (IFN)-based regimens have been the gold standard for treating HCV infection; this therapy is associated with a decreased risk of developing HCC [4–6]. With the recent development of direct-acting antivirals (DAAs), HCV treatment has become much easier because of the short-term and well-tolerated regimens, and a sustained virological response (SVR) is achieved in over 90% of patients.

Recent studies have reported that HCV-infected patients with a prior history of treated HCC and who subsequently achieved SVR with DAA therapies have an increased risk of HCC recurrence [7, 8]. Some studies have shown that the risks of early HCC recurrence after viral eradication are similar between IFN-based and DAA therapies [9, 10], and, furthermore, Waziry et al. performed a meta-analysis and reported that HCC recurrence was not different between IFN-based and DAA therapies [11]. However, even when HCC is completely removed with surgery or percutaneous ablation, it often recurs as intra-hepatic metastasis or multi-centric occurrence. HCC sometimes recurs even after achieving viral eradication with both IFN-based therapy and DAA therapy.

A past study reported that the HCC recurrence rates from the initiation of DAA treatment were 9.6–23.0% at 6 months and 23.1–45.7% at 12 months [12]. Most patients with a history of HCC therapy experienced HCC recurrence relatively early (within about 1 year) after DAA therapy. Moreover, the characteristics of patients with HCC recurrence in the early phase and late phase after completion of IFN-based or DAA treatment may be different [13, 14]. We hypothesized that, in the early phase after the end of anti-HCV treatment, the carcinogenic potential of the liver before beginning anti-HCV therapy may have a strong effect on HCC recurrence after anti-HCV treatment. However, in the late phase, the effects of other unknown risk factors may be present. Nevertheless, no studies have investigated different risk factors depending on time in one cohort.

In this study, we investigated HCC recurrence and risk factors for HCC recurrence after DAA treatment for HCV infection, and we clarified the different risk factors for early phase recurrence and late phase recurrence of HCC after the end of DAA therapy.

## Methods

### Patients

In this study, we retrospectively reviewed the medical records of 1663 consecutive patients diagnosed with HCV genotype 1 or 2 infection who were treated between 2014 and 2019 with IFN-free DAA regimens at 10 hospitals in the Ehime Kan-en Network (EKEN net). The 10 hospitals were Ehime University Hospital, Matsuyama Red Cross Hospital, Ehime Prefectural Central Hospital, Uwajima City Hospital, Saiseikai Imabari Hospital, Matsuyama Shimin Hospital, Ehime Prefectural Imabari Hospital, Ehime Prefectural Niihama Hospital, Saiseikai Matsuyama Hospital, and National Hospital Organization Ehime Medical Center. Of these patients, 199 patients had a previous history of HCC. Ninety of these 199 patients received 12 weeks of sofosbuvir and ledipasvir therapy, 26 received 12 weeks of sofosbuvir and ribavirin therapy, 53 received 24 weeks of daclatasvir and asunaprevir therapy, 18 received 12 weeks of ombitasvir, paritaprevir, and ritonavir therapy, and 12 received 12 weeks of elbasvir and grazoprevir therapy. We excluded patients who did not have a history of HCC before starting DAA therapy. All patients were confirmed to have no residual HCC prior to the start of DAA therapy by helical dynamic computed tomography (CT) or magnetic resonance imaging (MRI).

We excluded patients receiving warfarin at the start of DAA treatment from the analysis of prothrombin activity (PT %). We defined diabetes mellitus as patients with HbA1c > 6.5% or receiving anti-diabetes drugs or insulin before beginning DAA therapy.

### Clinical and laboratory assessments

Prior to DAA treatment, clinical and laboratory tests were performed. The Roche COBAS® TaqMan® HCV Auto assay system (Roche Molecular Diagnostics, Pleasanton, CA), which has a lower limit of detection of 1.2 log_10_ IU/mL, was used to determine HCV RNA levels. To quantitate liver fibrosis, we calculated the FIB-4 index (age (years) × (aspartate aminotransferase (AST)) [IU/L]/(platelet count [10^9^/L] × (alanine aminotransferase (ALT) [IU/L])^1/2^)) and APRI (AST to platelet ratio index; AST (/upper limit of normal) × 100/platelet count [10^9^/L]) as surrogate markers [15, 16]. These parameters were measured again on completion of DAA treatment and assessed in this study as post-treatment factors.

### Follow-up and diagnosis of HCC

Patients treated with DAAs were monitored every 3 to 6 months, including assessment of biochemical and virological values and blood counts, as well as screening for HCC with ultrasonography, helical dynamic CT, or MRI. For monitoring HCC recurrence, the selection of imaging modalities was at the discretion of the attending physician, with no specific protocol. The presence of typical hypervascular characteristics on angiography, as well as findings on dynamic CT or MRI, was used to diagnose HCC. If typical findings of HCC were not present, fine-needle aspiration biopsy and histological examination were performed to diagnose HCC.

HCC recurrence was defined as ‘early recurrence’ if it was within 1 year after DAA treatment completion and as ‘late recurrence’ if it was more than 1 year after DAA treatment completion.

### Statistical analysis

Significant differences were assessed with the χ^2^-test, Student’s *t*-test, or Welch’s *t*-test, as appropriate. Non-normally distributed parameters were evaluated with Welch’s *t*-test.

Cox proportional hazard model analyses were used to assess predictors of HCC recurrence following DAA therapy. Significant predictors that contributed to the recurrence of HCC on univariate analysis were inputted into the multivariate analysis. Hazard ratios (HRs) and 95% confidence intervals (CIs) were also calculated. All *p*-values < 0.05 on two-tailed testing were considered significant. The HCC recurrence rate was calculated using the Kaplan-Meier method. Differences in the rates of new HCC were tested with the log-rank test. Data were analyzed statistically using SPSS software ver. 23 (IBM, Armonk, NY, USA).

## Results

### Patients’ characteristics

We enrolled 126 males and 73 females with a median age at treatment initiation of 72 years (Table 1). The platelet count before DAA therapy was 11.9 ± 8.0 × 10^4^/μL, and α-fetoprotein (AFP) before DAA therapy was 20.8 ± 53.0 ng/mL (means ± standard deviation). Of the 199 patients, 184 (92%) achieved SVR following DAA therapy. At the start of DAA therapy, most patients (80.9%) were Child-Pugh class A. The BCLC stages of enrolled patients [17] at the latest HCC treatment were 81/49/31/35/3 (0/A/B/C/D), respectively.

**Table 1:** Clinical and virological characteristics of patients with a history of HCC treatments before and after DAA therapy

Age (years)	71.7 ± 8.0
Sex (male/female)	126/73
Body mass index (kg/m^2^)	23.2 ± 3.2
White blood cell count (/μL)	4295 ± 1370
Platelet count (× 10^4^/μL)	11.9 ± 8.0
ALT (U/L)	47.1 ± 30.3
AST (U/L)	52.3 ± 26.7
Total bilirubin (mg/dL)	0.8 ± 0.4
Albumin (g/dL)	3.8 ± 0.4
Prothrombin time (%)	82.9 ± 15.4
AFP (ng/mL)	20.8 ± 53.0
eGFR (mL/min/1.73 m^2^)	67.7 ± 20.3
Total cholesterol (mg/dL)	151.5 ± 27.9
Diabetes mellitus (no/yes)	149/49
Alcohol (none/drinking/unknown)	152/22/25
FIB-4 index	5.8 ± 3.7
APRI	1.6 ± 1.5
HCV RNA (log copies/mL)	5.8 ± 0.7
Post-treatment ALT (U/L)	22.8 ± 22.4
Post-treatment AST (U/L)	29.4 ± 16.4
Post-treatment total bilirubin (mg/dL)	0.8 ± 0.4
Post-treatment albumin (g/dL)	3.9 ± 0.4
Post-treatment prothrombin time (%)	83.5 ± 15.8
Post-treatment AFP (ng/mL)	8.2 ± 10.5
Post-treatment eGFR (mL/min/1.73 m2)	65.6 ± 19.3
SVR/no SVR	184/15
Child-Pugh class (A/B/C/unknown)	161/26/1/11
BCLC stage (0/A/B/C/D)	81/49/31/35/3
Latest HCC treatment (RFA/surgical resection)	178/21
Number of months from HCC treatment to DAA therapy initiation	20 ± 26
Number of curative treatments for HCC	1.1 ± 1.5
DAA therapy (SOF + LDV/SOF + RBV/ASV + DCV/OBV + PTV + r/EBR + GZR)	90/26/53/18/12

Data are expressed as means ± standard deviation

*ALT* alanine aminotransferase; *AST* aspartate aminotransferase; *AFP* α-fetoprotein; *eGFR* estimated glomerular filtration rate; *FIB-4* fibrosis-4; *APRI* AST to platelet ratio index; *HCV* hepatitis C virus; *RNA* ribonucleic acid; *SVR* sustained virological response; *BCLC* Barcelona Clinic Liver Cancer; *RFA* radiofrequency ablation; *DAA* direct-acting antiviral; *SOF* sofosbuvir; *LDV* ledipasvir; *RBV* ribavirin; *ASV* asunaprevir; *DCV* daclatasvir; *OBV* ombitasvir; *PTV* paritaprevir; *r* ritonavir; *EBR* elbasvir; *GZR* grazoprevir

### HCC recurrence rate

**Fig. 1:**
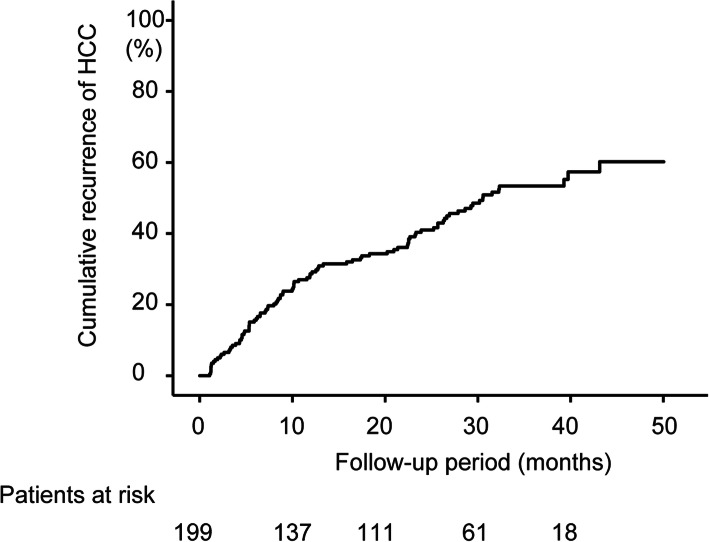
Cumulative recurrence (%) of hepatocellular carcinoma (HCC) after DAA therapy by Kaplan-Meier method

The median follow-up time after completion of DAA therapy was 22 months. Ninety-seven patients experienced HCC recurrence during the study period. The incidences of recurrence were 9.0, 16.6, 29.8, 41.0, and 53.4% at 4 and 6 months, and at 1, 2, and 3 years, respectively, after completion of DAA treatment (Fig. 1). Figure 1 also shows the numbers of patients followed up at each time point.

In 97 patients with HCC recurrence, the median time to recurrence was 10 months after the end of DAA treatment. We defined HCC recurrence within 1 year after DAA treatment completion as ‘early recurrence’, and that more than 1 year after DAA treatment as ‘late recurrence’, as described in the methods section.

### Survival rate after DAA therapy

Fourteen patients died during the study period. The incidences of death were 1.0, 2.6, and 6.4% at 1, 2, and 3 years, respectively, after completion of DAA treatment (Fig. S1A). Fig. S1 also shows the numbers of patients followed up at each time point. The survival rate was not significantly different between patients with and without SVR by the log-rank test (*p* = 0.46, Fig. S1B). On the other hand, the survival rate was significantly lower in patients with than in those without HCC recurrence (*p* = 0.026, Fig. S1C).

### Predictors of HCC recurrence after the end of DAA treatment in the overall observation period

For the overall period, we assessed parameters present before and after treatment that may have contributed to HCC recurrence after completion of DAA treatment, as shown in Tables 2 and 3.

**Table 2:** Factors associated with the recurrence of HCC after DAA treatment in the overall period

	HCC recurrence	No HCC recurrence	Hazard ratio	95% CI	p-value
Age (years)	71.9 ± 8.1	71.5 ± 8.1	1.00	0.98–1.03	0.71
Sex (male/female)	70/27	56/46	1.70	1.09–2.66	0.013
Body mass index (kg/m^2^)	23.1 ± 3.0	23.3 ± 3.5	0.98	0.92–1.05	0.79
White blood cell count (/μL)	4359 ± 1529	4235 ± 1207	1.00	0.99–1.00	0.52
Platelet count (×10^4^/μL)	12.3 ± 10.5	11.4 ± 4.6	1.00	0.98–1.02	0.43
ALT (U/L)	48.9 ± 32.5	45.4 ± 28.2	1.00	0.99–1.00	0.42
AST (U/L)	53.5 ± 25.7	51.1 ± 27.6	1.00	0.99–1.00	0.52
Total bilirubin (mg/dL)	0.8 ± 0.4	0.8 ± 0.4	0.96	0.61–1.52	0.89
Albumin (g/dL)	3.7 ± 0.4	3.8 ± 0.4	0.68	0.42–1.11	0.35
Prothrombin time (%)	83.3 ± 13.9	82.6 ± 16.8	1.00	0.99–1.01	0.78
AFP (ng/mL)	22.5 ± 58.8	19.1 ± 46.8	1.00	0.99–1.00	0.65
eGFR (mL/min/1.73 m^2^)	66.1 ± 19.5	69.1 ± 21.0	0.99	0.98–1.00	0.31
Total cholesterol (mg/dL)	151 ± 29.0	151 ± 26.8	1.00	0.99–1.00	0.96
Diabetes mellitus (no/yes)	68/28	81/21	1.43	0.92–1.00	0.18
Alcohol (none/drinking/unknown)	69/12/21	83/10/4	0.90	0.59–1.38	0.18
FIB-4 index	5.9 ± 3.8	5.7 ± 3.6	1.01	0.96–1.07	0.62
APRI	1.6 ± 1.5	1.5 ± 1.5	1.04	0.92–1.16	0.56
Post-treatment white blood cell count (/μL)	4845 ± 1434	4599 ± 1576	1.00	0.99–1.00	0.26
Post-treatment ALT (U/L)	21.3 ± 10.3	24.1 ± 29.4	0.99	0.97–1.00	0.39
Post-treatment AST (U/L)	28.8 ± 12.3	29.9 ± 19.5	0.99	0.98–1.00	0.64
Post-treatment total bilirubin (mg/dL)	0.8 ± 0.4	0.8 ± 0.3	0.98	0.60–1.58	0.72
Post-treatment albumin (g/dL)	3.9 ± 0.4	3.9 ± 0.3	0.76	0.48–1.21	0.56
Post-treatment prothrombin time (%)	83.2 ± 14.1	83.9 ± 17.2	1.00	0.98–1.01	0.76
Post-treatment AFP (ng/mL)	9.7 ± 13.0	6.4 ± 6.3	1.03	0.99–1.05	0.11
Post-treatment eGFR (mL/min/1.73 m^2^)	63.0 ± 18.7	67.9 ± 19.8	0.98	0.97–1.00	0.10
SVR/no SVR	86/11	98/4	2.55	1.35–4.80	0.048
Child-Pugh class (A/B/C)	78/13/1	83/13/0	1.17	0.68–2.02	0.58
BCLC stage (0/A/B/C/D)	37/25/28/16/1	44/24/13/19/2	1.06	0.90–1.25	0.75
Number of months from HCC treatment to DAA therapy initiation	17 ± 25	23 ± 26	1.00	0.99–1.00	0.12
Number of curative treatments for HCC	1.4 ± 1.6	0.8 ± 1.3	1.23	1.09–1.38	0.004

**Table 3:** Independent factors associated with the recurrence of HCC after DAA treatment in the overall period according to Cox proportional hazard model analysis

	Hazard ratio	95% CI	p-value
Male	1.75	1.09–2.80	0.019
No SVR	2.30	1.17–4.52	0.015
Number of curative treatments for HCC	1.21	1.07–1.36	0.001

Data are expressed as means ± standard deviation

*ALT* alanine aminotransferase; *AST* aspartate aminotransferase; *AFP* α-fetoprotein; *eGFR* estimated glomerular filtration rate; *FIB-4* fibrosis-4; *APRI* AST to platelet ratio index; *SVR* sustained viral response; *BCLC* Barcelona Clinic Liver Cancer; *CI* confidence interval

Results of univariate analyses are shown in Table 2. Multivariate analysis identified three factors, male sex (hazard ratio (HR) = 1.75; 95% CI 1.09–2.80, *p* = 0.019), no SVR achievement (HR = 2.30; 95% CI 1.17–4.52, *p* = 0.015), and number of treatments for HCC (HR = 1.21; 95% CI 1.07–1.36, *p* = 0.001), as independent factors that contributed to the recurrence of HCC (Table 3).

**Fig. 2:**
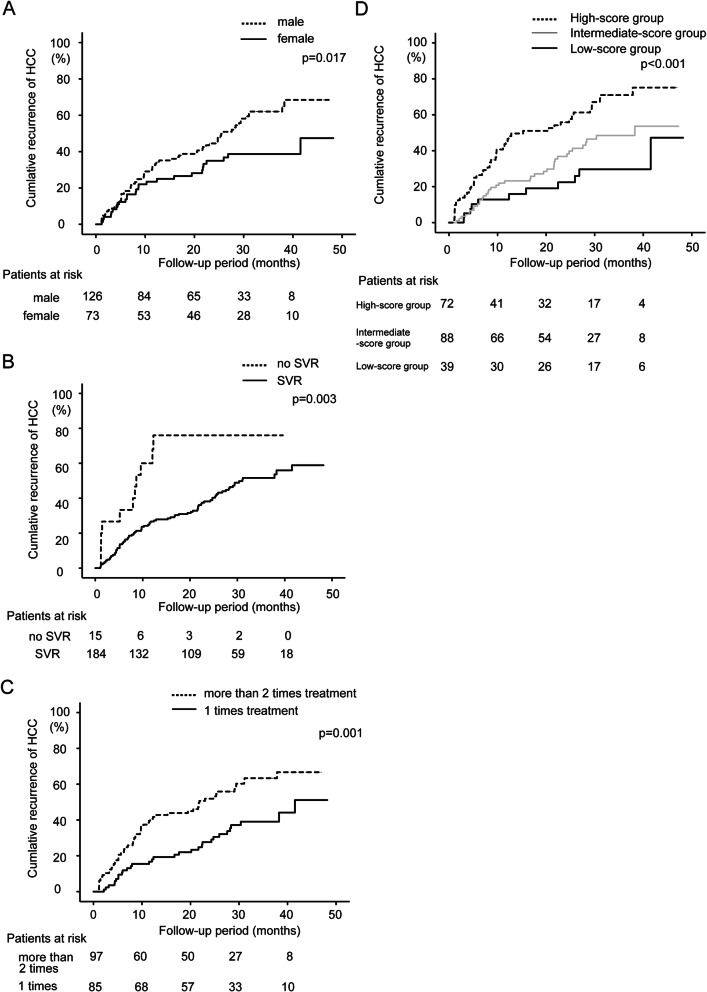
Comparison of cumulative HCC recurrence (%) by sex (**A**). Cumulative HCC recurrence is significantly higher in males than in females, according to the log-rank test (p = 0.017). Comparison of cumulative HCC recurrence (%) by sustained virological response (SVR) achievement (**B**). Cumulative HCC recurrence is significantly higher in the ‘no SVR’ group than in the group with SVR according to the log-rank test (p = 0.003). Comparison of cumulative HCC recurrence (%) between the group with more than two past HCC treatments and the group with only one HCC treatment (**C**). Cumulative HCC recurrence is significantly higher in the group with more than two past HCC treatments according to the log-rank test (p = 0.001). Cumulative recurrence (%) of HCC according to the score combining sex, SVR achievement, and number of past HCC treatments (**D**). Study patients are grouped based on these scores: 0 points, low-risk group (*n* = 39); 1 or 2 points, intermediate-risk group (*n* = 88); and 3 points, high-risk group (*n* = 72). Cumulative HCC recurrence increases significantly with higher scores (*p* < 0.001)

The recurrence of HCC was examined according to the factors that contributed to the recurrence of HCC after DAA treatment on multivariate analysis (sex, SVR/no SVR, and number of HCC treatments). Cumulative HCC recurrence was significantly higher in male patients than in female patients on the log-rank test (*p* = 0.017, Fig. 2a). Similarly, cumulative HCC recurrence was significantly higher in patients without SVR than with SVR (*p* = 0.003, Fig. 2b), and recurrence was significantly higher in patients with a history of more than two treatments for HCC than in those with only one treatment for HCC (*p* = 0.001, Fig. 2c).

### Recurrence of HCC stratified by a score that combined sex, SVR achievement, and number of HCC treatments

Based on the results of multivariate analysis, a scoring system that combined sex, SVR achievement, and the number of HCC treatments was developed. Female and male sex were scored as 0 and 1, respectively. Patients with and without SVR were scored as 0 and 1, respectively. Patients with only one treatment and two or more treatments for HCC were scored 0 and 1, respectively.

Of the study patients, 39, 88, 66, and 6 had scores of 0, 1, 2, and 3, respectively. The study patients were then grouped based on these scores as follows: 0 points, low-risk group (*n* = 39); 1 or 2 points, intermediate-risk group (*n* = 88); and 3 points, high-risk group (*n* = 72). Figure 2d shows the cumulative incidence curve of HCC recurrence for each group. The 1- and 2-year cumulative incidence rates of HCC recurrence were 12.8 and 22.4% in the low-risk group, 23.2 and 36.8% in the intermediate-risk group, and 46.6 and 55.8% in the high-risk group, respectively. Cumulative HCC recurrence increased significantly with higher scores (*p* < 0.001, Fig. 2d).

### Predictors of HCC recurrence after the end of DAA treatment in the early phase

**Fig. 3:**
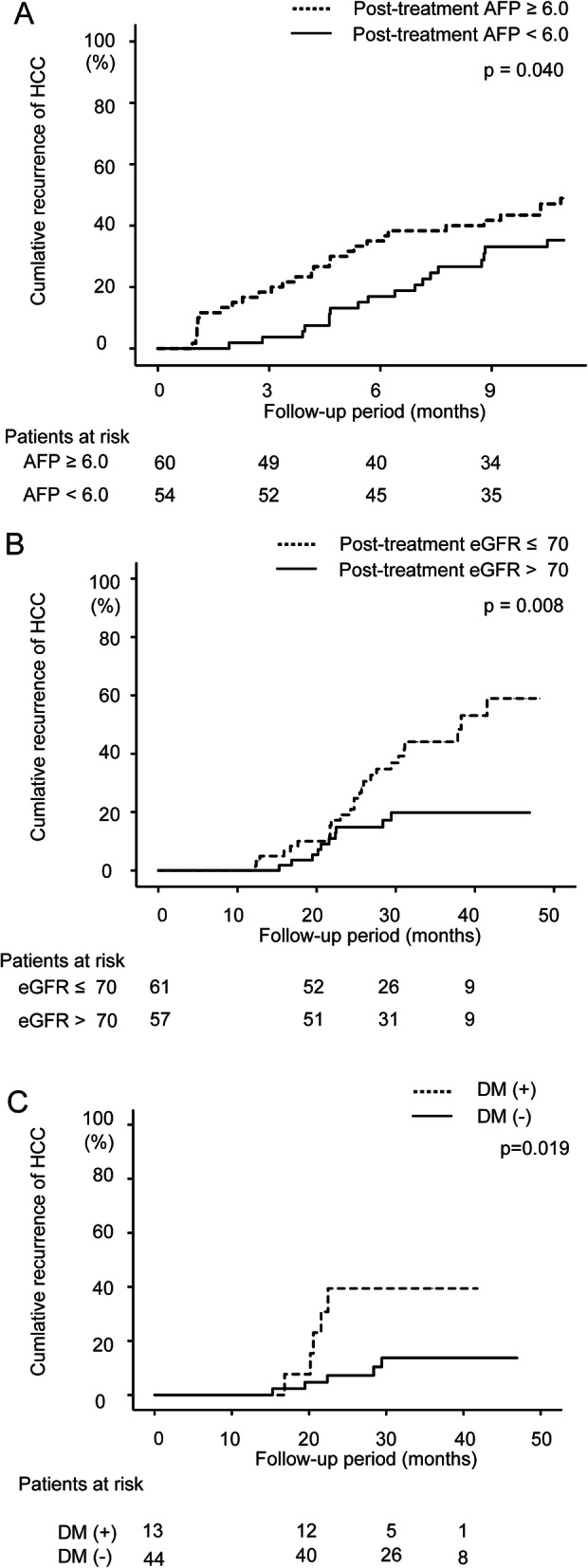
Comparison of cumulative HCC recurrence (%) in the early phase (within 1 year after DAA treatment) by post-treatment AFP ≥6.0 ng/mL (**A**). Cumulative HCC recurrence in the early phase is significantly higher in the high-AFP group than in the low-AFP group according to the log-rank test (*p* = 0.040). Comparison of cumulative HCC recurrence (%) in the late phase (more than 1 year after DAA treatment) by post-treatment eGFR ≤70 mL/min/1.73 m^2^ (**B**). Cumulative HCC recurrence in the late phase is significantly higher in the low-eGFR group than in the high-eGFR group according to the log-rank test (*p* = 0.008). Comparison of cumulative HCC recurrence (%) in the late phase in patients with post-treatment eGFR > 70 mL/min/1.73 m^2^ between groups with and without diabetes mellitus (DM) (**C**). Cumulative HCC recurrence in the late phase is significantly higher in the group with DM than in the group without DM according to the log-rank test (*p* = 0.019)

Pre-treatment factors and post-treatment factors that may have contributed to ‘early recurrence’ after the end of DAA treatment were evaluated. Potential predictors associated with HCC recurrence were the same as those in the analysis of the overall period. Results of univariate analyses are shown in Table 4. Multivariate analysis identified post-treatment AFP (HR = 1.056; 95% CI 1.026–1.087, p < 0.001) as an independent factor that contributed to the recurrence of HCC (Table 5). The log-rank test showed that cumulative HCC recurrence was significantly higher in patients whose post-treatment AFP was ≥6.0 ng/mL than in patients whose post-treatment AFP was < 6.0 ng/mL (*p* = 0.040, Fig. 3a). Optimal cut-off values were set in accordance with receiver operating characteristic curve analysis (Fig. S2A). The area under the curve value for the post-treatment AFP was 0.63. The optimal cut-off value for the post-treatment AFP was 6.0 ng/mL, with sensitivity of 57.4% and specificity of 54.0%.

**Table 4:** Factors associated with the recurrence of HCC after DAA treatment in the early phase (within 1 year after DAA treatment completion)

	HCC recurrence	No HCC recurrence	Hazard ratio	95% CI	p-value
Age (years)	70.8 ± 8.6	71.5 ± 8.1	0.99	0.96–1.02	0.63
Sex (male/female)	41/17	56/46	1.71	0.97–3.02	0.064
Body mass index (kg/m^2^)	23.1 ± 2.8	23.3 ± 3.5	0.99	0.91–1.08	0.79
White blood cell count (/μL)	4309 ± 1464	4235 ± 1207	1.00	0.99–1.00	0.72
Platelet count (×10^4^/μL)	10.7 ± 5.1	11.4 ± 4.6	0.97	0.91–1.03	0.36
ALT (U/L)	51.0 ± 36.2	45.4 ± 28.2	1.00	0.99–1.01	0.28
AST (U/L)	55.4 ± 27.7	51.1 ± 27.6	1.00	0.99–1.01	0.35
Total bilirubin (mg/dL)	0.8 ± 0.4	0.8 ± 0.4	1.14	0.64–2.00	0.79
Albumin (g/dL)	3.7 ± 0.4	3.8 ± 0.4	0.56	0.30–1.04	0.10
Prothrombin time (%)	82.1 ± 12.8	82.6 ± 16.8	0.99	0.98–1.01	0.82
AFP (ng/mL)	29.3 ± 74.1	19.1 ± 46.8	1.00	0.99–1.00	0.30
eGFR (mL/min/1.73 m^2^)	66.8 ± 20.5	69.1 ± 21.0	0.99	0.98–1.00	0.50
Total cholesterol (mg/dL)	150 ± 32.1	151 ± 26.8	0.99	0.98–1.00	0.92
Diabetes mellitus (no/yes)	42/16	81/21	1.44	0.81–2.56	0.33
Alcohol (none/drinking/unknown)	51/4/3	69/12/21	0.68	0.33–1.39	0.29
FIB-4 index	6.6 ± 4.4	5.7 ± 3.6	1.04	0.99–1.10	0.33
APRI	1.9 ± 1.8	1.5 ± 1.5	1.09	0.98–1.21	0.33
Post-treatment white blood cell count (/μL)	4764 ± 1421	4599 ± 1576	1.00	0.99–1.00	0.52
Post-treatment ALT (U/L)	21.2 ± 9.2	24.1 ± 29.4	0.99	0.97–1.01	0.47
Post-treatment AST (U/L)	29.1 ± 9.8	29.9 ± 19.5	0.99	0.97–1.01	0.76
Post-treatment total bilirubin (mg/dL)	0.9 ± 0.4	0.8 ± 0.3	1.15	0.61–2.18	0.65
Post-treatment albumin (g/dL)	3.8 ± 0.5	3.9 ± 0.3	0.63	0.34–1.16	0.29
Post-treatment prothrombin time (%)	82.5 ± 14.3	83.9 ± 17.2	0.99	0.98–1.01	0.62
Post-treatment AFP (ng/mL)	11.9 ± 15.5	6.4 ± 6.3	1.04	1.02–1.06	0.028
Post-treatment eGFR (mL/min/1.73 m^2^)	63.5 ± 18.2	67.9 ± 19.8	0.99	0.97–1.00	0.19
SVR/no SVR	48/10	98/4	2.85	1.43–5.64	0.007
Child-Pugh class (A/B/C)	46/9/1	83/13/0	1.46	0.77–2.75	0.37
BCLC stage (0/A/B/C/D)	18/16/14/10/0	44/24/13/19/2	1.10	0.89–1.37	0.22
Number of months from HCC treatment to DAA therapy initiation	12 ± 14	23 ± 26	0.99	0.99–0.99	0.001
Number of curative treatments for HCC	1.7 ± 1.7	0.8 ± 1.3	1.25	1.10–1.41	0.001

**Table 5:** Independent factors associated with the recurrence of HCC after DAA treatment in the early phase (within 1 year after DAA treatment completion) according to Cox proportional hazard model analysis

	Hazard ratio	95% CI	p-value
Post-treatment AFP (ng/mL)	1.056	1.026–1.087	< 0.001

Data are expressed as means ± standard deviation

*ALT* alanine aminotransferase; *AST* aspartate aminotransferase; *AFP* α-fetoprotein; *eGFR* estimated glomerular filtration rate; *FIB-4* fibrosis-4; *APRI* AST to platelet ratio index; *SVR* sustained viral response; *BCLC* Barcelona Clinic Liver Cancer; *CI* confidence interval

### Predictors of HCC recurrence after the end of DAA treatment in the late phase

Pre-treatment factors and post-treatment factors that may have contributed to ‘late recurrence’ after the end of DAA treatment were evaluated. Potential predictors associated with HCC recurrence were the same as those in the analysis of the overall period. Results of univariate analyses are shown in Table 6. Multivariate analysis identified post-treatment post-treatment estimated glomerular filtration rate (eGFR) (HR = 0.98; 95% CI 0.96–0.99, *p* = 0.032) as an independent factor that contributed to the recurrence of HCC (Table 7). The log-rank test showed that cumulative HCC recurrence was significantly higher in patients whose post-treatment eGFR was ≤70 mL/min/1.73 m^2^ than in patients whose post-treatment eGFR was > 70 mL/min/1.73 m^2^ (*p* = 0.008, Fig. 3b). Optimal cut-off values were set in accordance with receiver operating characteristic curve analysis (Fig. S2B). The area under the curve value for the post-treatment eGFR was 0.64. The optimal cut-off value for the post-treatment eGFR was 70 mL/min/1.73 m^2^, with a sensitivity of 57.3% and specificity of 72.2%. To clarify the features of patients whose post-treatment eGFR was ≤70 mL/min/1.73 m^2^, factors associated with eGFR ≤70 mL/min/1.73 m^2^ at the end of DAA treatment were evaluated. Age (*p* = 0.017) and the number of curative treatments for HCC (*p* = 0.021) were associated with eGFR ≤70 mL/min/1.73 m^2^ at the end of DAA treatment (Table S1). Patients with eGFR ≤70 mL/min/1.73 m^2^ at the end of DAA treatment were often those with multiple past HCC treatments.

**Table 6:** Factors associated with the recurrence of HCC after DAA treatment in the late phase (more than 1 year after DAA treatment completion)

	HCC recurrence	No HCC recurrence	Hazard ratio	95% CI	p-value
Age (years)	73.5 ± 7.0	70.6 ± 8.1	1.04	0.99–1.08	0.062
Sex (male/female)	29/10	48/39	2.15	1.04–4.42	0.049
Body mass index (kg/m^2^)	23.1 ± 3.2	23.3 ± 3.6	0.97	0.88–1.07	0.79
White blood cell count (/μL)	4435 ± 1641	4311 ± 1250	1.00	0.99–1.00	0.64
Platelet count (×10^4^/μL)	14.8 ± 15.2	11.4 ± 4.6	1.01	0.99–1.03	0.064
ALT (U/L)	45.6 ± 26.1	46.7 ± 29.1	0.99	0.98–1.00	0.84
AST (U/L)	50.7 ± 22.3	52.2 ± 28.5	0.99	0.98–1.00	0.76
Total bilirubin (mg/dL)	0.8 ± 0.4	0.8 ± 0.4	0.75	0.35–1.60	0.80
Albumin (g/dL)	3.8 ± 0.4	3.8 ± 0.4	1.05	0.48–2.28	0.79
Prothrombin time (%)	85.1 ± 15.5	81.6 ± 16.6	1.01	0.99–1.03	0.28
AFP (ng/mL)	12.2 ± 14.8	17.6 ± 40.9	0.99	0.97–1.01	0.43
eGFR (mL/min/1.73 m^2^)	65.1 ± 18.3	71.6 ± 19.4	0.98	1.00–1.02	0.020
Total cholesterol (mg/dL)	152 ± 23	151 ± 26	1.00	0.99–1.02	0.85
Diabetes mellitus (no/yes)	26/12	70/17	1.77	0.89–3.51	0.16
Alcohol (none/drinking/unknown)	32/6/1	62/12/3	1.29	0.74–2.25	0.23
FIB-4 index	4.9 ± 2.5	5.7 ± 3.7	0.89	0.78–1.01	0.24
APRI	1.2 ± 0.7	1.5 ± 1.6	0.65	0.41–1.02	0.24
Post-treatment white blood cell count (/μL)	4964 ± 1465	4710 ± 1606	1.00	0.99–1.00	0.41
Post-treatment ALT (U/L)	21.5 ± 11.9	25.0 ± 31.6	0.99	0.97–1.01	0.52
Post-treatment AST (U/L)	28.4 ± 15.5	30.5 ± 20.6	0.98	0.96–1.01	0.58
Post-treatment total bilirubin (mg/dL)	0.8 ± 0.4	0.8 ± 0.4	0.78	0.35–1.73	0.98
Post-treatment albumin (g/dL)	3.9 ± 0.4	3.9 ± 0.3	1.03	0.48–2.21	0.61
Post-treatment prothrombin time (%)	84.3 ± 13.9	82.8 ± 17.3	1.00	0.98–1.02	0.67
Post-treatment AFP (ng/mL)	6.0 ± 5.3	6.6 ± 6.6	0.97	0.90–1.05	0.69
Post-treatment eGFR (mL/min/1.73 m^2^)	62.7 ± 19.6	70.6 ± 17.0	0.98	0.96–0.99	0.013
SVR/no SVR	38/1	84/3	0.78	0.10–5.73	1.00
Child-Pugh class (A/B/C)	32/4/0	71/12/0	0.68	0.24–1.93	0.77
BCLC stage (0/A/B/C/D)	19/9/4/6/1	39/20/12/15/1	0.97	0.74–1.28	0.94
Number of months from HCC treatment to DAA therapy initiation	25 ± 34	21 ± 23	1.00	0.99–1.00	0.51
Number of curative treatments for HCC	1.0 ± 1.2	0.8 ± 1.4	1.13	0.89–1.43	0.44

**Table 7:** Independent factors associated with the recurrence of HCC after DAA treatment in the late phase (more than 1 year after DAA treatment completion) according to Cox proportional hazard model analysis

	Hazard ratio	95% CI	p-value
Post-treatment eGFR (mL/min/1.73 m^2^)	0.98	0.96–0.99	0.032

Data are expressed as means ± standard deviation

*ALT* alanine aminotransferase; *AST* aspartate aminotransferase; *AFP* α-fetoprotein; *eGFR* estimated glomerular filtration rate; *FIB-4* fibrosis-4; *APRI* AST to platelet ratio index; *SVR* sustained viral response; *BCLC* Barcelona Clinic Liver Cancer; *CI* confidence interval

Even among patients with post-treatment eGFR > 70 mL/min/1.73 m^2^, 10 patients had late recurrence of HCC after DAA treatment. We evaluated the factors associated with no recurrence of HCC in the late phase (more than 1 year after DAA treatment completion) in patients with eGFR > 70 mL/min/1.73 m^2^ at the end of DAA treatment. The absence of diabetes mellitus was identified as a factor that contributed to no recurrence in the late phase in patients with post-treatment eGFR > 70 mL/min/1.73 m^2^ (*p* = 0.038, Table S2). On the log-rank test, the cumulative incidence of late HCC recurrence was significantly higher in patients with diabetes mellitus than in those without diabetes mellitus in patients with post-treatment eGFR > 70 mL/min/1.73 m^2^ (*p* = 0.019, Fig. 3c).

## Discussion

Previous studies have examined parameters that may predict HCC recurrence after DAA therapy. The cumulative incidence of HCC recurrence is lower in patients who achieve SVR compared to those who do not [9, 18, 19]. Guarino et al. reported a significant inverse relationship between SVR and risk of HCC recurrence [12]. Thus, achieving SVR is an important parameter to predict future HCC recurrence.

The HR was 2.22 for HCC recurrence after DAA therapy in patients with previous HCC recurrence compared to those without previous HCC recurrence (only one treatment for HCC [20]. Thus, a prior history of HCC recurrence before DAA therapy also affected HCC recurrence after DAA [18, 20, 21].

In the present study, independent factors associated with the recurrence of HCC after DAA treatment in the overall period according to Cox proportional hazard model analyses were male sex, no SVR, and the number of curative treatments for HCC. Although SVR achievement and the number of curative treatments were similar to the previous reports described above, male sex had not been reported. HCC is a male-dominant cancer [22], and irrespective of the etiology, rates of HCC are two to four times higher in men than in women. Several studies have reported that males are at high risk of developing HCC after IFN-based therapy [23, 24]. In the present results, sex difference also affected HCC recurrence after IFN-free DAA regimens [25]. As in some previous reports, in the present study, absence of SVR was strongly associated with recurrence after DAA therapy. Thus, an analysis was conducted excluding patients who did not achieve SVR. However, both in the early phase and the late phase, the risk factors for recurrence of HCC after DAA therapy were the same as those including patients without SVR (data not shown).

We identified one independent factor, post-treatment AFP, that was associated with early recurrence of HCC. Serum AFP levels are often increased in patients with HCC, and thus, the AFP level is commonly used as a surrogate marker for HCC [26]. In patients with IFN-based therapy, post-treatment AFP levels are significantly associated with HCC occurrence, and higher AFP levels, even after these treatments, are associated with a higher risk of developing HCC [27, 28]. In patients undergoing DAA treatment, we recently reported that a higher FIB-4 index and higher post-treatment AFP are independent risk factors for de novo HCC development (without previous HCC treatment patients) [29]. In addition, post-treatment AFP is an independent factor associated with early recurrence of HCC within 6 months after antiviral therapy [13]. In our analysis of the overall period, the AFP level was not identified as an independent factor, and thus, a higher level of post-treatment AFP may be a specific risk factor associated with HCC recurrence in the early phase.

On the other hand, in late phase recurrence, post-treatment eGFR ≤70 mL/min/1.73 m^2^ was the only independent risk factor identified in this study. This factor has not been reported in past studies, but post-treatment eGFR ≤70 mL/min/1.73 m^2^ was associated with the number of past HCC treatments (Supplemental Table 1), which was a risk factor for HCC recurrence after DAA treatment. Some patients with a post-treatment eGFR > 70 mL/min/1.73 m^2^ experienced HCC recurrence in the late phase after DAA treatment. In patients with post-treatment eGFR > 70 mL/min/1.73 m^2^, those without diabetes mellitus had a lower risk of HCC recurrence than those with diabetes mellitus (Supplemental Table 2). In IFN therapy, abnormalities in glucose metabolism are significantly associated with HCC development [24]. Recently, Degasperi et al. reported that diabetes is independently associated with HCC recurrence in patients who received DAA treatment [30].

BCLC stage was determined by tumor status, liver function, and performance status. This study included 38 patients with BCLC stage C or D who were expected to have a poor prognosis and might not be candidates for curative treatments such as radiofrequency ablation or surgical resection. However, since all of these patients had no vascular invasion or extrahepatic metastases, and their performance status was ≥1, they were considered eligible for curative treatment by their treating physicians. Therefore, BCLC stage was not identified as a significant factor for recurrence in this study.

The HCC recurrence rate may depend on the period after DAA treatment [12], and most patients with previous HCC treatment had experienced HCC recurrence in a relatively early time. Patients with early recurrence may have already had invisible HCC before DAA treatment, and, thus, factors associated with HCC were predictive. On the other hand, after a period of time, these factors may not have an effect on late recurrence, because the effect of factors associated with HCC before treatment is attenuated. Therefore, the characteristics of patients with HCC recurrence in the early phase may be different from those with recurrence in the late phase after completion of DAA treatment. However, before our report, few studies had clearly shown differences in risk factors according to the period after the end of DAA treatment.

## Conclusions

In conclusion, risk factors associated with HCC recurrence after DAA therapies are different in the early (within 1 year after DAA treatment) and late phases. Patients with a higher level of post-treatment AFP within 1 year, and after 1 year, those with a history of more than two treatments for HCC or those with diabetes mellitus, which are related to post-treatment eGFR, require careful attention regarding the possibility of HCC recurrence.

## Supplementary Information

**Additional file 1: Supplementary Figure 1.** The survival rate (%) after DAA therapy by Kaplan-Meier method (A). Comparison of survival rate (%) by sustained virological response (SVR) achievement (B). The survival rate is not significantly different between patients with and without SVR according to the log-rank test. Comparison of survival rate (%) by HCC recurrence after DAA therapy (C). The survival rate is significantly lower in the group with HCC recurrence than in the group without HCC recurrence after DAA therapy according to the log-rank test (*p* = 0.026).

**Additional file 2: Supplementary Figure 2.** Receiver operating characteristic (ROC) curves for the post-treatment AFP to predict HCC recurrence in early phase (within 1 year after DAA treatment). The area under the curve (AUC) value for the post-treatment eGFR is 0.63 (A). Receiver operating characteristic (ROC) curves for the post-treatment eGFR to predict HCC recurrence in late phase (more than 1 year after DAA treatment). The area under the curve (AUC) value for the post-treatment eGFR is 0.64 (B).

**Additional file 3.** Supplementary Table 1. Factors associated with eGFR ≤70 mL/min/1.73 m^2^ at the end of DAA treatment.

**Additional file 4.** Supplementary Table 2. Factors associated with no HCC recurrence in the late phase (more than 1 year after DAA treatment completion) among patients with eGFR > 70 mL/min/1.73 m^2^ at the end of DAA treatment.

## Data Availability

The datasets used and/or analysed during the current study are available from the corresponding author on reasonable request.

## Abbreviations

HCV: Hepatitis C virus
HCC: Hepatocellular carcinoma
IFN: Interferon
DAAs: Direct-acting antivirals
SVR: Sustained virological response
CT: Computed tomography
MRI: Magnetic resonance imaging
PT: Prothrombin activity
AST: Aspartate aminotransferase
ALT: Alanine aminotransferase
HR: Hazard ratio
CI: Confidence interval
AFP: α-fetoprotein
eGFR: estimated glomerular filtration rate
