# Supplementary figures and images for: AFP and eGFR are related to early and late recurrence of HCC following antiviral therapy
Source: BMC Cancer. 2021 Jun 14;21:699. doi: 10.1186/s12885-021-08401-7 (PMC8201700; doi:10.1186/s12885-021-08401-7)

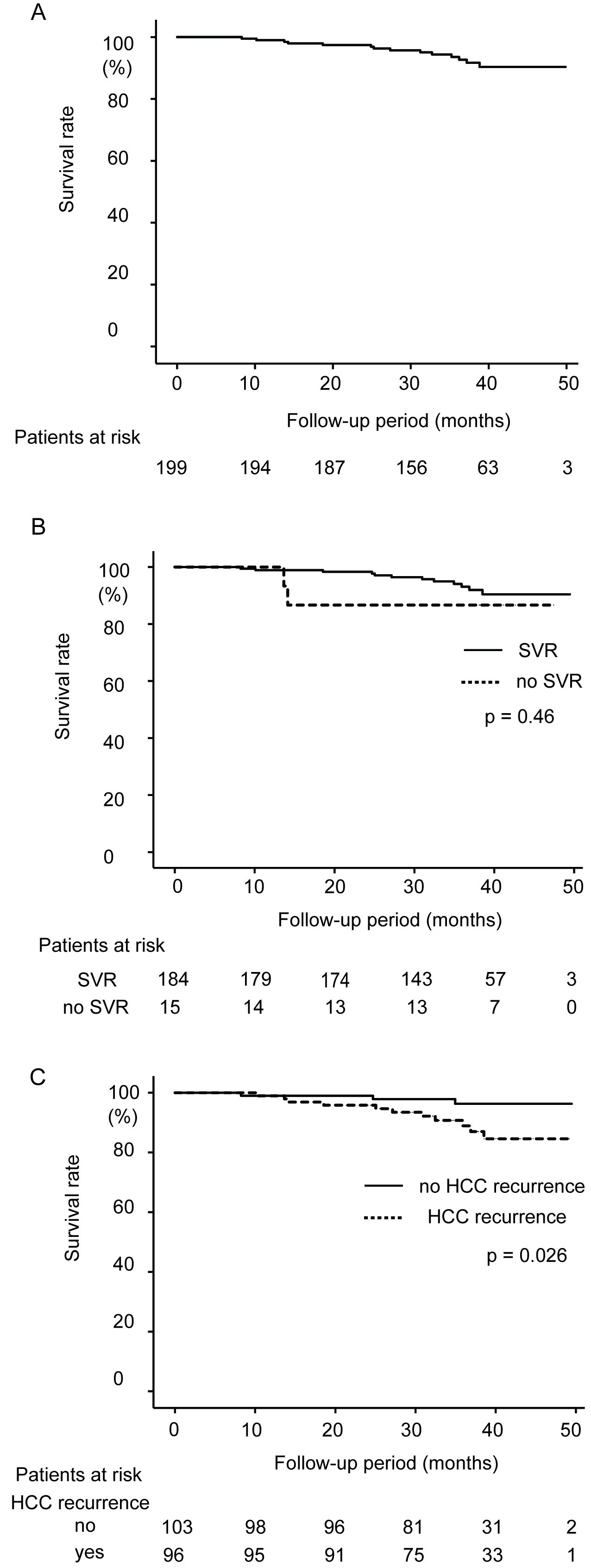

Supplement: Supplementary file 1 — Additional file 1: Supplementary Figure 1. The survival rate (%) after DAA therapy by Kaplan-Meier method (A). Comparison of survival rate (%) by sustained virological response (SVR) achievement (B). The survival rate is not significantly different between patients with and without SVR according to the log-rank test. Comparison of survival rate (%) by HCC recurrence after DAA therapy (C). The survival rate is significantly lower in the group with HCC recurrence than in the group without HCC recurrence after DAA therapy according to the log-rank test (p = 0.026). [file 12885_2021_8401_MOESM1_ESM.tif]

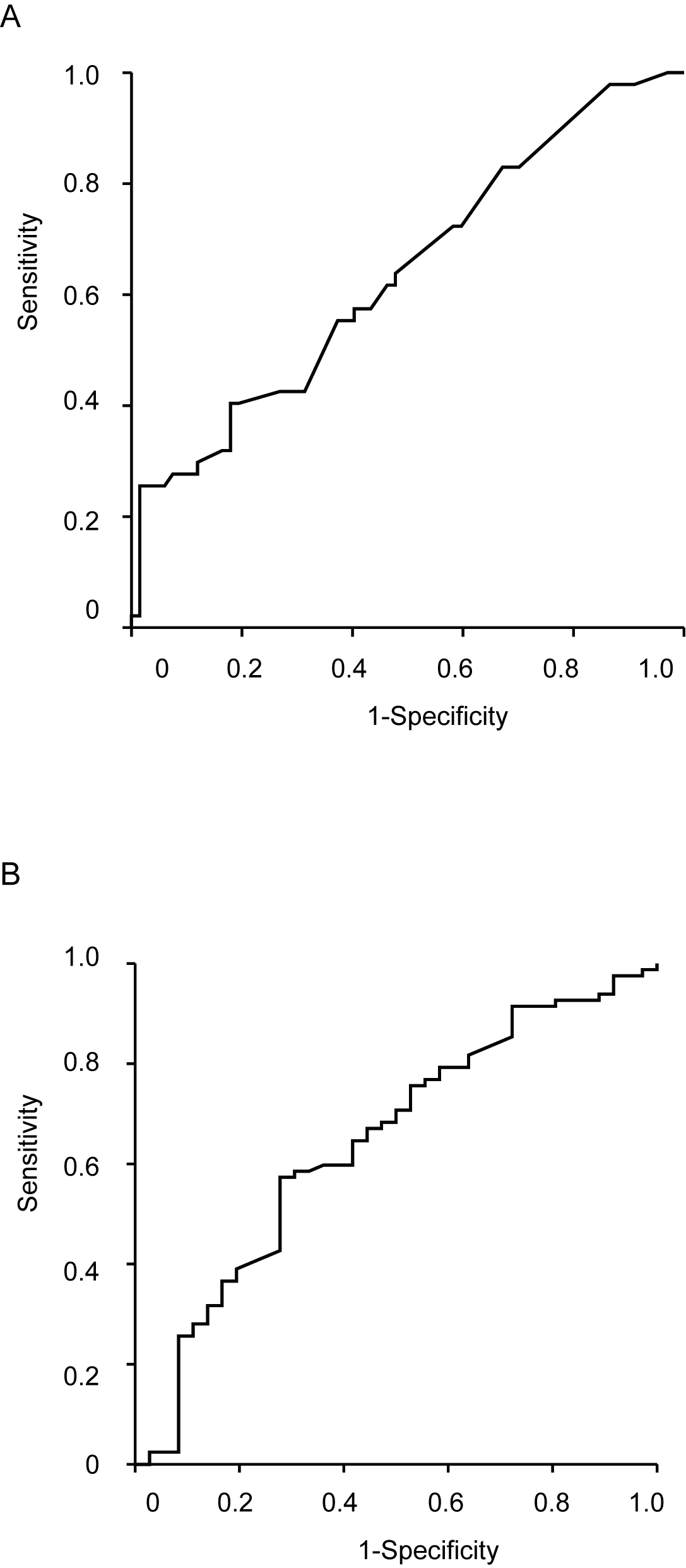

Supplement: Supplementary file 2 — Additional file 2: Supplementary Figure 2. Receiver operating characteristic (ROC) curves for the post-treatment AFP to predict HCC recurrence in early phase (within 1 year after DAA treatment). The area under the curve (AUC) value for the post-treatment eGFR is 0.63 (A). Receiver operating characteristic (ROC) curves for the post-treatment eGFR to predict HCC recurrence in late phase (more than 1 year after DAA treatment). The area under the curve (AUC) value for the post-treatment eGFR is 0.64 (B). [file 12885_2021_8401_MOESM2_ESM.tif]
