# Supplementary material for: AFP and eGFR are related to early and late recurrence of HCC following antiviral therapy
Source: BMC Cancer. 2021 Jun 14;21:699. doi: 10.1186/s12885-021-08401-7 (PMC8201700; doi:10.1186/s12885-021-08401-7)
Supplement: Supplementary file 3 — Additional file 3. Supplementary Table 1. Factors associated with eGFR ≤70 mL/min/1.73 m2 at the end of DAA treatment. [file 12885_2021_8401_MOESM3_ESM.docx]

Supplementary Table 1.

Factors associated with eGFR ≤ 70 mL/min/1.73 m^2^ at the end of DAA treatment

|  | eGFR ≤ 70 | eGFR > 70 | p-value |
| --- | --- | --- | --- |
| Age (years) | 73 ± 7.5 | 69.8 ± 8.2 | 0.017 |
| Sex (male/female) | 39/22 | 32/25 | 0.45 |
| Body mass index (kg/m^2^) | 23.4 ± 3.5 | 23.3 ± 3.6 | 0.88 |
| White blood cell count (/µL) | 4257 ± 1319 | 4357 ± 1419 | 0.69 |
| Platelet count (×10^4^/µL) | 11.4 ± 4.0 | 12.1 ± 5.1 | 0.41 |
| ALT (U/L) | 41.4 ± 23.0 | 50.2 ± 30.9 | 0.082 |
| AST (U/L) | 48.1 ± 20.5 | 55.2 ± 31.7 | 0.15 |
| Total bilirubin (mg/dL) | 0.8 ± 0.3 | 0.8 ± 0.5 | 0.75 |
| Albumin (g/dL) | 3.8 ± 0.4 | 3.9 ± 0.3 | 0.44 |
| Prothrombin time (%) | 80.0 ± 16.0 | 83.9 ± 15.7 | 0.20 |
| AFP (ng/mL) | 18.6 ± 47.6 | 13.1 ± 15.5 | 0.41 |
| Total cholesterol (mg/dL) | 152 ± 24.4 | 151 ± 26.7 | 0.90 |
| Diabetes mellitus (no/yes) | 45/15 | 44/13 | 0.83 |
| FIB-4 index | 5.5 ± 2.4 | 5.6 ± 4.4 | 0.78 |
| APRI | 1.3 ± 0.7 | 1.6 ± 1.9 | 0.22 |
| Post-treatment white blood cell count (/µL) | 4863 ± 1602 | 4741 ± 1523 | 0.67 |
| Post-treatment ALT (U/L) | 21.7 ± 15.3 | 26.3 ± 36.2 | 0.36 |
| Post-treatment AST (U/L) | 29.0 ± 16.7 | 30.9 ± 21.9 | 0.59 |
| Post-treatment total bilirubin (mg/dL) | 0.8 ± 0.3 | 0.9 ± 0.4 | 0.21 |
| Post-treatment albumin (g/dL) | 3.9 ± 0.4 | 3.9 ± 0.3 | 0.28 |
| Post-treatment prothrombin time (%) | 81.1 ± 15.8 | 86.1 ± 16.7 | 0.11 |
| Post-treatment AFP (ng/mL) | 6.4 ± 7.4 | 6.5 ± 4.6 | 0.96 |
| SVR/no SVR | 58/3 | 57/0 | 0.24 |
| Number of curative treatment for HCC | 1.2 ± 1.6 | 0.6 ± 0.9 | 0.021 |

Data are expressed as means ± standard deviation.

eGFR, estimated glomerular filtration rate; ALT, alanine aminotransferase; AST, aspartate aminotransferase; AFP, α-fetoprotein; FIB-4, fibrosis-4; APRI, AST to platelet ratio index; SVR, sustained viral response
