# Supplementary material for: AFP and eGFR are related to early and late recurrence of HCC following antiviral therapy
Source: BMC Cancer. 2021 Jun 14;21:699. doi: 10.1186/s12885-021-08401-7 (PMC8201700; doi:10.1186/s12885-021-08401-7)
Supplement: Supplementary file 4 — Additional file 4. Supplementary Table 2. Factors associated with no HCC recurrence in the late phase (more than 1 year after DAA treatment completion) among patients with eGFR > 70 mL/min/1.73 m2 at the end of DAA treatment. [file 12885_2021_8401_MOESM4_ESM.docx]

Supplementary Table 2.

Factors associated with no HCC recurrence in the late phase (more than 1 year after DAA treatment completion) among patients with eGFR > 70 mL/min/1.73 m^2^ at the end of DAA treatment

|  | HCC | No HCC | p-value |
| --- | --- | --- | --- |
| Age (years) | 70.2 ± 7.2 | 69.7 ± 8.5 | 0.88 |
| Sex (male/female) | 7/3 | 25/22 | 0.48 |
| Body mass index (kg/m^2^) | 22.1 ± 2.6 | 23.6 ± 3.8 | 0.27 |
| White blood cell count (/µL) | 4585 ± 2197 | 4308 ± 1223 | 0.58 |
| Platelet count (×10^4^/µL) | 14.0 ± 4.8 | 11.7 ± 5.1 | 0.19 |
| ALT (U/L) | 39.3 ± 18.9 | 52.5 ± 32.6 | 0.22 |
| AST (U/L) | 46.9 ± 23.3 | 57.0 ± 33.1 | 0.36 |
| Total bilirubin (mg/dL) | 0.7 ± 0.4 | 0.8 ± 0.5 | 0.45 |
| Albumin (g/dL) | 3.8 ± 0.2 | 3.9 ± 0.4 | 0.65 |
| Prothrombin time (%) | 84.3 ± 17.4 | 83.8 ± 15.5 | 0.92 |
| AFP (ng/mL) | 10.7 ± 12.0 | 13.6 ± 16.3 | 0.59 |
| Total cholesterol (mg/dL) | 146 ± 20.2 | 153 ± 28.4 | 0.52 |
| Diabetes mellitus (no/yes) | 5/5 | 39/8 | 0.038 |
| Alcohol (none/drinking/unknown) | 8/2 | 30/7/10 | 0.19 |
| FIB-4 index | 4.2 ± 2.1 | 5.9 ± 4.7 | 0.26 |
| APRI | 1.1 ± 0.6 | 1.8 ± 2.1 | 0.30 |
| Post-treatment white blood cell count (/µL) | 5251 ± 2096 | 4632 ± 1378 | 0.24 |
| Post-treatment ALT (U/L) | 18.3 ± 10.4 | 28.0 ± 39.4 | 0.44 |
| Post-treatment AST (U/L) | 24.9 ± 8.7 | 32.1 ± 23.7 | 0.34 |
| Post-treatment total bilirubin (mg/dL) | 0.8 ± 0.5 | 0.9 ± 0.4 | 0.66 |
| Post-treatment albumin (g/dL) | 3.8 ± 0.3 | 4.0 ± 0.3 | 0.27 |
| Post-treatment prothrombin time (%) | 83.1 ± 14.3 | 86.7 ± 17.2 | 0.56 |
| Post-treatment AFP (ng/mL) | 7.3 ± 8.5 | 6.3 ± 2.8 | 0.73 |
| Number of curative treatments for HCC | 1.0 ± 1.1 | 0.5 ± 0.9 | 0.21 |

Data are expressed as means ± standard deviation.

eGFR, estimated glomerular filtration rate; ALT, alanine aminotransferase; AST, aspartate aminotransferase; AFP, α-fetoprotein; FIB-4, fibrosis-4; APRI, AST to platelet ratio index
